# Identifying, understanding and responding to domestic abuse in the perinatal period

**DOI:** 10.1192/bjb.2023.19

**Published:** 2024-06

**Authors:** Lucy Peacock, Imrana Puttaroo, Bo Kim Tang, Alex B. Thomson

**Affiliations:** 1 London North West University Healthcare NHS Trust, London, UK; 2 West London NHS Trust, London, UK; 3 Central and North West London NHS Foundation Trust, London, UK

**Keywords:** Risk assessment, perinatal psychiatry, education and training, consent and capacity, psychosocial interventions

## Abstract

Domestic abuse often begins or escalates during the perinatal period, increasing the risk of adverse pregnancy outcomes and death of the woman and infant. The hidden nature of domestic abuse, compounded by barriers to disclosure, means many clinicians are likely to have unknowingly encountered a patient who is being abused and missed a vital opportunity for intervention. This educational article presents the experience of a woman who was abused during pregnancy. It describes how to facilitate a disclosure and conduct an assessment and illustrates safeguarding duties alongside interventions.

## Clinical scenario

### Patient's perspective

‘I reached out for help when I was 6 months pregnant and thought things couldn't get worse. My emotions were impossible to deal with and I was crying every day. My relationship with my partner was turbulent but from the outside we were the perfect couple. He always told me what we were going through was normal and all relationships have ups and downs, he said we needed to work together. Our domestic problems began with arguments about money or his infidelity, which he denied. He became angry and threatening and blamed me for his behaviour. I spoke to my friend, who suggested professional support, but it was difficult to talk about details, even with people close to me, because I felt so embarrassed. When I saw the doctor, I understood they were trying to help but I didn't see how they could. We spoke about my mood, but I didn't feel able to tell them about my home life.

After the birth I went back to the doctor because having a baby changed my perspective on my relationship. I realised that I wasn't just thinking about myself and had to consider my child's welfare. I left my partner and moved in with my family, but my mood was still low, and I knew that I needed help to process what had happened.

The situation between me and my partner throughout my pregnancy became more and more volatile. The first time he hit me was after he had been pressuring me to help him pay off a loan. We argued and he knocked me into the wardrobe. I packed my things and left. The days following, he called me repeatedly, degrading me and telling me that no one would want me. Eventually we got back together. When he found out I was pregnant he kicked and strangled me.

Opening up to professionals enabled me to get the help I needed for me and my child. In the early days I still felt so guilty as I chose this man to be my partner and he ended up being abusive and fathering my child. Now I can reflect things happen in life which aren't great, but the crucial thing is that there is an accessible service with trained people to speak to who are removed from the situation personally and can offer help.’

### Doctor's perspective

You are a junior doctor working in the community mental health team. You have a follow-up appointment with a 24-year-old woman who had been referred by her general practitioner with persistent low mood throughout her pregnancy, against a background of social stressors. The first time you saw her, she spoke quietly and avoided eye contact. You noticed some bruising on her arms. You remember she was anxious to leave and her partner rang her repeatedly during the consultation. She told you that he was wondering why she had been so long. On direct questioning, she said that ‘everything is fine at home’.

You are glad she has attended this follow-up appointment as you had previously identified some warning signs that she might have been experiencing domestic abuse. Today she attends your clinic alone and discloses that her former partner, from whom she has since separated, had been progressively manipulative and emotionally and physically abusive throughout her pregnancy.

Box 1 lists some questions to consider.

Box 1 Questions to consider regarding domestic abuse in the perinatal period

- What are the warning signs of domestic abuse in pregnancy?
- How would you facilitate a disclosure?
- How would you proceed with a safe risk assessment and management?
- What consideration will you give to the pregnancy in the context of domestic abuse?
- Which members of the multidisciplinary team could you seek advice from?
- What are the responsibilities of mental health professionals in supporting victims and survivors of domestic abuse?

### Ethics, consent and terminology

As this is an educational article that does not report original research no ethics review was needed. Written informed consent was obtained from the patient for the publication of clinical details. The patient's perspective was transcribed and summarised from an interview.

This article uses the term ‘women’, which encompasses both cis and trans women. We are aware that parents could have any gender identity and could also be cis, trans, non-binary and/or intersex.

## Discussion

### The impact of domestic abuse in the perinatal period

The Domestic Abuse Act 2021 defines domestic abuse as any act of abusive behaviour between personally connected people aged 16 years or over. The Act clarifies that ‘abusive behaviour’ could encompass isolated or repeated incidents of physical or sexual abuse, threatening, controlling or coercive behaviour, financial control and emotional abuse (e.g. belittling, manipulating and gaslighting.) People who are ‘personally connected’ could be intimate partners, ex-partners, family members or individuals who share parental responsibility for a child. There is no requirement for the victim and perpetrator to live in the same household.^1^

Rates of domestic abuse are higher against people with mental disorders.^2,3^ Increasing evidence has found that people with severe mental illness are at high risk of being victims of violence in general, with 40–90% experiencing violence over a lifetime.^4^ Pregnancy is a time when new mental disorders can develop and pre-existing ones can relapse, making pregnant women with mental disorders particularly vulnerable to experiencing domestic abuse. Thirty per cent of domestic abuse starts during pregnancy; where domestic abuse has already existed in a relationship, pregnancy causes its escalation. There is a high correlation between antenatal and postnatal violence.^5^

In some cases, the nature of domestic abuse may change during pregnancy as the perpetrator may make a conscious effort not to hurt the developing baby; instead, they will increase their controlling and emotionally abusive behaviours.^6^ Covert forms of psychological aggression (such as raised voices and insults) have been found to have a significant impact during pregnancy.^7^ It should be noted that the process of pregnancy and the birth itself are profound events that can be challenging for victims of sexual abuse.^8^

Reports from MBBRACE-UK (Mothers and Babies: Reducing Risk through Audits and Confidential Enquiries across the UK) regularly investigate the causes of maternal deaths across the UK. A maternal death is defined as a woman who has died during pregnancy or up to 1 year after the end of pregnancy.

The prevalence of reported domestic abuse in England is 3.4% during pregnancy and 5.8% during the postnatal period.^9^ This is likely to be a gross underestimate, as MBBRACE-UK reports often state that data on domestic abuse are missing when reviewing cases of maternal deaths posthumously. Twelve per cent of the women who died during or up to a year after pregnancy in the UK in 2018–2020 reported being a victim of domestic abuse during their pregnancy.^10^

Consecutive MBBRACE-UK reports have noted that women at ‘severe disadvantage’ are consistently over-represented among maternal deaths in the UK. The main elements of ‘severe disadvantage’ are a psychiatric diagnosis, substance misuse and being a victim of domestic abuse. Of the 495 maternal deaths in the UK between 2017 and 2019, 40 (8%) were of women considered to have ‘multiple severe disadvantages’ and all but 3 of these women had experienced domestic abuse.^11^

There are specific implications for an infant of a woman exposed to domestic abuse during pregnancy and these are outlined in Box 2.^12,13^ It cannot be assumed that preverbal infants are unaware and thus unaffected by domestic abuse taking place. There is a 2.5 times increased risk of infant death during pregnancy when domestic abuse occurs, with a common cause of death being blows to the woman's abdomen.^14^ Domestic abuse during pregnancy is specifically associated with low birth weight and behavioural problems in the child at 42 months of age.^15^

Box 2 Implications for the infant exposed to domestic abuse *in utero*^12,13^

Short term:

- higher rates of preterm birth and low birth weights
- increased risk (6-fold) of both perinatal and neonatal death

Long term:

- difficult temperament in early childhood
- physical aggression, disobeying rules, cheating, stealing or destruction of property and internalising symptoms (anxiety or depression) during later childhood

Longer-term neurodevelopmental effects on the infant can be explained by the increased levels of cortisol produced by pregnant women enduring the stress of domestic abuse. Increased cortisol levels interrupt the development of the fetus's own neurodevelopmental system. This can manifest in the infant having more difficulty regulating their emotions in childhood.^16,17^

### Facilitating a disclosure of domestic abuse

Routine enquiry about domestic abuse is recommended in both perinatal and general mental health settings. Even with routine enquiry, detection of cases falls short of the true prevalence of domestic abuse in people in contact with healthcare services.^18^ This is partly attributable to barriers perceived or experienced by patients which prevent them from disclosing domestic abuse to healthcare professionals.

A qualitative study^21^ identified fear as a key theme preventing disclosure and these concerns should not be trivialised (Box 3). If disclosure ultimately leads to a decision to leave an abusive relationship, this can expose the victim to a higher risk of lethal violence.^19^ Forty-one per cent of women killed by a partner/former partner in the UK in 2018 had taken steps to separate from them; nearly two-thirds of these women were killed within the first year of separation.^20^

Box 3 Specific concerns preventing the disclosure of domestic abuse^21^

- Social services involvement
- Concerns that the disclosure will not be believed
- Further violence from the perpetrator
- Losing financial support of the perpetrator
- Disruption to family life
- Consequences for their immigration status
- Feelings of self-blame, shame and embarrassment

There are many factors relating to the context of the consultation and skills of the clinician that can also be barriers to disclosure. A victim of domestic abuse may encounter multiple intersectional barriers related to victim-blaming and perceived lack of credibility, as a result of others’ assumptions about gender, ethnicity, sexuality or status as a person with a mental disorder.^22–24^ People with mental disorders are often seen as less credible.^25^ They may have difficulty expressing themselves, anticipate that they may not be believed, or judge that disclosure may increase the risk of abuse or reprisal. It is vital not to automatically assume that a disclosure of domestic abuse is part of a person's mental illness – remember that both domestic abuse and pregnancy can cause deterioration in a person's mental state.

Many pregnant women who experience domestic abuse do not seek professional help.^26^ There are specific patterns of behaviour and warning signs that can help prompt you to further enquire about domestic abuse during pregnancy (Box 4).

Box 4 Warning signs that domestic abuse may be present in pregnancy^27,28^

- Traumatic injury (particularly repeated injury accompanied by vague explanations)
- Intrusive partner during consultations
- Repetitive genitourinary symptoms
- Vaginal bleeding or sexually transmitted infections
- Increased alcohol use and substance misuse
- Depression and anxiety
- Self-harm and suicidal ideation
- Adverse reproductive outcomes (including multiple unintended pregnancies, recurrent terminations, premature labour and stillbirth)
- Non-attendance or late booking of antenatal reviews
- Poor nutrition and inadequate gestational weight gain

To increase the likelihood that a victim will disclose domestic abuse to you, ensure that a private, face-to-face setting is offered. Although family and carer involvement in healthcare is important, ensure that patients are given the opportunity to speak without a third party present. With the use of virtual appointments increasing following the COVID-19 pandemic, the patient cannot be assumed to be alone and the perpetrator may be able to listen in on the appointment.

A good working knowledge of issues related to domestic abuse will mean that you appear more understanding, credible and trustworthy to a victim. This includes knowledge of patterns of perpetrator behaviour, experiences and dilemmas faced by victims, discrimination and structural barriers to seeking help and awareness of relevant local services. Victims are more likely to disclose domestic abuse if you use open-ended questions to initiate the topic and use sensitive, validating follow-up questions and statements (Box 5). The project Linking Abuse and Recovery through Advocacy for Victims and Perpetrators (LARA-VP) has produced resources that outline multiple methods for exploring domestic abuse within a clinical consultation.^5^

Box 5 Examples of screening questions and validating statements

*‘Because domestic abuse is so common, particularly in pregnancy, we always ask patients about it.’*

*‘Tell me about how you met your partner. What is your relationship like?’*

*‘Have you ever felt pressured by your partner into doing something you didn't want to do?’*

*‘Do you ever feel unsafe or frightened with your partner? Have they ever been unkind to you?’*

*‘How do you manage conflict as a couple?’*

*‘When you said that your partner would loom over you, it sounds like you were really scared.’*

*‘You mentioned you used your body to shield the baby. It sounds like you were doing the best you could to protect the baby.’*

*‘It sounds like you were being very brave in that moment.’*

### Management following disclosure

One of the most important tests of a therapeutic relationship comes when sensitively handling a disclosure of domestic abuse, as the victim has traversed significant barriers to get to this point. The process involves a delicate balance between working with the victim to make changes to protect themselves and any children and continually reviewing risk.

You first need to be able to assess their immediate safety.^27^ Useful lines of questioning will establish whether the abuse is ongoing, whether there is anyone else at home, the location of the perpetrator and of any other children, whether there are any active threats and whether the victim has a safe place to stay.

Victims who are at very high risk of serious harm need immediate referral to a multi-agency risk assessment conference (MARAC). In these conferences, representatives from different professional bodies discuss high-risk domestic abuse cases with the desired outcome of an action plan that will safeguard the victims. Pregnancy and giving birth within the past 18 months are MARAC high-risk indicators.^29^

A more detailed history of the abuse is needed, focusing on the MARAC high-risk indicators (Box 6). Risk assessment tools should not be used purely to generate a risk score, but rather as a means of gathering contextual information for areas of risk that should be enquired about. If cause for concern is identified, you should discuss this with your team and line manager. The SafeLives Domestic Abuse Stalking and Harassment (DASH) checklist is a tool to help identify victims who are at very high risk of harm and therefore need MARAC referral.^29^

Box 6 MARAC high-risk indicators^29^

- Injury
- Fear of further injury or violence
- Isolation from family and friends
- Depressed mood and/or suicidal thoughts
- Attempted separation
- Conflict over child contact
- Constant attempts at contact by the perpetrator
- Pregnant or given birth within the past 18 months
- Increased frequency or worsening seriousness of abuse
- Coercive control
- Excessive jealousy
- Use of weapons/objects to cause harm
- Threats to kill victim, children or others

Provide a clear pathway of the next steps and do not rely on the victim to self-refer to other services. Focus on empowering the victim. Immediate action may be needed: a victim who is asking for help should never be discharged to an unsafe environment with the assumption that a referral to social services will be sufficient to protect them. Seek advice and do not act alone. Principles of safe enquiry and locally agreed safeguarding processes should be used to establish the level of risk posed to the victim.^30^ Risk is dynamic; assess and manage risk on an ongoing basis, and consistently document what has and has not been done.

When a pregnant woman discloses domestic abuse, there are other services and multidisciplinary team members you can seek specific advice from:

- perinatal mental health services – to assess and manage women with mental illness during the perinatal period; they will play an important role in monitoring the victim's mental state and treatment adherence while continually assessing risk
- obstetricians and midwives – to help form birth plans for complex pregnancies affected by domestic abuse
- health visitors
- social services.

### Confidentiality and capacity

Aim to work collaboratively with victims by reassuring them that help is available. Confidentiality should be emphasised, but the limits of this should be made clear. For example, if a vulnerable third party such as a child is at risk, a disclosure to further services may have to be made without consent.^30^ Be transparent about why you believe information should be shared. Be clear that sharing information with social services will not necessarily mean removal of their child after birth and it is intended as a safeguarding intervention.

Always inform a victim in advance about planned information sharing, unless there is a compelling reason not to do so.^31^ Where you are considering sharing information without consent, you should refer to professional guidance and seek advice from your Caldicott guardian and safeguarding lead. The General Medical Council has issued specific guidance on disclosing information about people who may be at risk of harm.^31,32^ Victims should be encouraged to be involved in decisions about disclosing their personal information – if they are declining to do this, try to explore their specific reasons for doing so. Support and empower them to make decisions in their own interests – for example by arranging contact with agencies to support victims of domestic abuse.^31^

A victim's ‘capacity’ to decide to stay in a relationship with a domestic abuse perpetrator, in the context of coercive control and real threats, is a problematic concept. Capacity assessments should never be used as a justification for not helping a victim.^33^ Capacity is a dynamic concept; a victim who initially refuses consent to sharing information with others is not necessarily refusing all forms of help, and may change their mind later on as trust in you develops.

### Safeguarding interventions

Where imminent risk to the victim is identified this should be discussed with a local independent domestic violence advocate (IDVA), a local domestic abuse lead or the 24 h National Domestic Abuse Helpline (www.nationaldahelpline.org.uk). IDVAs are specialist domestic abuse professionals who support victims of domestic abuse and will prioritise their safety; they have in-depth knowledge of many areas, including the criminal justice processes and housing, financial and children's services.^29^ IDVA support is essential in practically helping a victim navigate these complex services as well as the emotional impact of domestic abuse.

As a clinician it is important to be up to date with local referral policies for your area of work. Although there may be a need for the involvement of the police, a victim may have valid reasons for not wanting this, and access to help should never be contingent on reporting to the police.

The interrelating aspects of domestic abuse and mental disorder means that your professional role extends beyond the development of a trusting relationship to facilitate a disclosure and the onward referrals to relevant services. This includes advocacy and the need to address structural discrimination, for example by clearly communicating to other relevant agencies that a victim's disclosure is not symptoms of a mental disorder and therefore must be taken seriously and acted on. Pregnant women are vulnerable to both being victims of domestic abuse and having an increased risk of developing mental disorders and it is your role to highlight this to other agencies.

During the postnatal period, women abruptly lose the wide breadth of services they accessed during pregnancy. Ongoing close liaison with relevant agencies should occur during the postnatal period to effectively safeguard the victim. Longer-term psychiatric treatment planning for the mother and child should also consider the psychological effects of having survived domestic abuse. Specific therapeutic interventions include trauma-focused cognitive–behavioural therapy, eye movement desensitisation and reprocessing and parent–child interventions.

### Reflection

As a clinician, be mindful of your own unconscious emotional reactions and countertransference towards patients. Your own personal biases, demographics and beliefs may affect your ability to recognise the warning signs of domestic abuse. Remember to consider domestic abuse in all patients and be especially vigilant in the perinatal period. You should respond sensitively to a disclosure while remaining curious about the patient's story in order to form a safe management plan.

Clinician behaviours remain vital to building a therapeutic relationship and facilitating a disclosure of domestic abuse within hectic clinical environments. Support for clinical issues and reflecting on your emotional response should be available in reflective practice, Balint groups and supervision sessions.

Your colleagues may have been affected by domestic abuse so be sensitive to this when working in multidisciplinary team settings. As clinicians caring for patients within the unique, high-risk perinatal setting, we must also recognise the potential impact of compassion fatigue and secondary trauma on ourselves and our colleagues.

## Conclusions

This article highlights the significant adverse outcomes and risks associated with domestic abuse during the perinatal period. Detection of domestic abuse and intervention during pregnancy is a vital opportunity to reduce the risks to both mother and infant. Pregnancy and early parenthood are periods when a woman will frequently encounter healthcare professionals; it is a duty of all clinicians to incorporate domestic abuse screening into their routine enquiries.

If you or someone you know has been affected by the issues raised in this article, the National Domestic Abuse Helpline can be contacted on 0808 2000 247 or at www.nationaldahelpline.org.uk.

## Data Availability

Data availability is not applicable to this article as no new data were created or analysed in this study.

## About the authors

**Lucy Peacock**, BSc, MBBS, is a foundation doctor at London North West University Healthcare NHS Trust, London, UK. **Imrana Puttaroo**, BMedSci, BMBS, MRCPsych, is an ST5 specialty registrar in general adult psychiatry at West London NHS Trust, London, UK. **Bo Kim Tang**, MBBS, BSc, MRCPsych, is a substantive consultant perinatal psychiatrist at Central and North West London NHS Foundation Trust, London, UK. **Alex B. Thomson**, BA, MBBS, MA, FRCPsych, is a consultant liaison psychiatrist at Central and North West London NHS Foundation Trust, London, UK.
